# Staff awareness of the use of cannabidiol (CBD): a trust-wide survey study in the UK

**DOI:** 10.1186/s42238-021-00104-x

**Published:** 2021-12-15

**Authors:** Obioha Ukaegbu, Jared Smith, David Hall, Thomas Frain, Cyrus Abbasian

**Affiliations:** 1 East Wandsworth Community Mental Health Team, Springfield Hospital, South West London and St George’s Mental Health NHS Trust, 61 Glenburnie Road, London, SW17 7DJ UK; 2 Population Health Research Institute, St George’s, University of London, Cranmer Terrace, Tooting, London, SW17 0RE UK

**Keywords:** Cannabidiol, Medical cannabis, Clinical staff, United Kingdom, Attitudes

## Abstract

**Introduction:**

Cannabidiol (CBD) is now a legal substance in Europe and is available in ‘high street shops’, usually as CBD oil. However, in the United Kingdom (UK), there is no clear consensus among healthcare professionals and organisations over how to manage CBD use in their patients. This is an important issue as CBD is a constituent of ‘medicinal and recreational cannabis’ and is gaining support in the scientific literature and lay media for use in physical and mental health problems. Given the aforementioned, this study is an exploration of healthcare professionals’ beliefs and attitudes with regard to CBD.

**Methods:**

In July 2018, we sent requests by email to approximately 2000 clinical staff (including 319 physicians) at a mental health trust in South West London to answer 8 questions in a single survey using Surveyplanet.com, about their beliefs regarding CBD. There was no specific method of choosing the staff, and the aim was to get the email request sent to as many staff as possible on each service line. We did an analysis to see how the attitudes and beliefs of different staff member groups compared. We also gave them space to offer free text responses to illustrate their ideas and concerns. We used chi-squared tests for comparison across groups and used odds ratio for pairwise group comparisons.

**Results:**

One hundred ninety surveys were received in response, and of these, 180 were included in the final sample. The physician response rate was 17.2% (55/319); the response rate for non-physicians could not be estimated as their total number was not known at outset. 32.2% of the responders had the right to prescribe (58/180) and 52.8% had an experience of working in addiction services (95/180).

We found that staff members who can prescribe were 1.99 times as likely to believe CBD has potential therapeutic properties compared to those who do not (*OR* = 1.99, *CI* = 1.03, 3.82; *p* = 0.038) and 2.94 times less likely to think it had dangerous side effects (*OR* = 0.34, *CI* = 0.15, 0.75; *p* = 0.006). Prescribing healthcare professionals were 2.3 times as likely to believe that CBD reduces the likelihood of psychosis (*OR* = 2.30, *CI* = 1.10, 4.78; *p* = 0.024). However, prescribing healthcare professionals with the ability to prescribe were 2.12 times as likely to believe that CBD should be prescription only (*OR* = 2.12, *CI* = 1.12, 4.01; *p* = 0.02). Individuals experienced in addiction services were 2.22 times as likely to be associated with a belief that CBD has therapeutic properties (*OR* = 2.22, *CI* = 1.22, 4.04; *p* = 0.009). Staff in general reported a lack of knowledge about CBD in their free text responses.

**Conclusions:**

With almost 95% of prescribers being physicians, they appear to demonstrate awareness of potential therapeutic benefit, reduced likelihood of psychosis and seeming lack of dangerous side effects with CBD. However, their higher stringency about the need for prescription implies an attitude of caution. There was also a suggestion that biases about cannabis were influencing responses to questions as well. The external validity of this study could be diminished by sampling bias and limitation to a single mental health trust. Nonetheless, some of the results drew a reasonable comparison with similar studies.

## Introduction

In the United Kingdom (UK), cannabis is known as a drug of abuse and it is classified as illegal as a class B drug under the UK’s Misuse of Drug Act 1971, where class A deemed as the most harmful and class C is deemed as the least. The maximum penalty for possession of a class B drug in the UK is 5-year imprisonment (Shiner 2015).

Some cannabis varieties are known to contain high quantities of a chemical compound called 9-tetrahydrocannabinol (THC) that is psychoactive and is associated with psychotic episodes (Di Forti et al. 2009).

However, some cannabis varieties produce significant amounts of another compound called cannabidiol (CBD) which can counter the effects of THC (Russo 2011; Aso et al. 2019). There is also growing evidence that CBD can reduce the severity of psychotic symptoms (Iffland and Grotenhermen 2017; Bhattacharyya et al. 2018; Davies and Bhattacharyya 2019).

CBD is not thought to significantly activate CB1 and CB2 receptors as THC does (Devinsky et al. 2014) and may reduce the psychoactive effects of THC by negative allosteric modulation at CB1 (Laprairie et al. 2015). However, it is thought to have other molecular targets including equilibrative nucleoside transporters (ENT), GPR55 receptors, TRPM8 channels, 5HT1a receptors, alpha1 and 3 glycine receptors, and TRPV1 and TRPV2 channels and an indirect effect on adenosine A1 receptor and has a bidirectional effect on intracellular calcium (Devinsky et al. 2014). Some of the actions in these areas are thought to include reduction of THC psychoactive activity and anti-spasticity effects in multiple sclerosis; though there is also a high degree of uncertainty around the effects and mechanism of these actions (Devinsky et al. 2014).

CBD has been noted to have anti-inflammatory and neuroprotective effects (Iffland and Grotenhermen 2017; Viar Fogaça et al. 2013). Isolated CBD has been increasingly recommended in the treatment of many conditions including epilepsy, multiple sclerosis (MS) and pain, for instance (Maroon and Bost 2018; O'Connell et al. 2017; Mücke et al. 2018); this can be in isolation or in combination with THC (Maroon and Bost 2018). Epilepsy and MS often have comorbidity with mental health conditions (Chwastiak and Ehde 2007; Hesdorffer et al. 2012), so there is a potential of mental health professionals coming into contact with CBD-based preparations in the future.

Products containing CBD and cannabinoids in general can be prescribed for specific indication by physicians who are registered on the United Kingdom General Medical Council’s (GMC) specialist register (Freeman et al. 2019). To be on this register, a physician has to be at the Consultant level which is the most senior position a physician can hold in the UK.

However, CBD is now available in Europe, without prescription at ‘high street shops’ so long as any THC contained in the preparation is less than 0.2% (Hughes, 2018). The term ‘high street’ is a reference to the major roads of any town or city. It is important to note that the typical doses of this ‘high street’ CBD are significantly lower than the concentrations found to be of therapeutic value in studies (McGregor et al. 2020). There are also doubts over the quality of the product (Freeman et al. 2019). CBD preparations in the shops are not the same as cannabis, which is usually available to the UK population illegally via drug dealers in the street varieties. The common street varieties in the UK are ‘cannabis resin’ (hashish) and ‘herbal cannabis’ (sinsemilla) (Potter et al. 2018). Sinsemilla typically has a higher THC content than resin and less CBD (Hardwick and King 2008). And since the 2000s, the illegal cannabis market in the UK has become increasingly dominated by sinsemilla to the point that it made up approximately 80% of police seizures of cannabis (Potter et al. 2018; Hardwick and King 2008). Therefore, the CBD preparations in shops bear very little relationship to cannabis that is sold illegally on the street.

Globally, the prescription-free trend for CBD is maintained and it is available without prescription in many states in the United States (US), where established pharmacy stores have sold products ranging from patches to creams (McGregor et al. 2020). Although officially there is no clear consensus in the legislature of US federal and state institutions, given that more CBD preparations are coming to market and clinical staff are more likely to be in contact with these substances, we decided to do a survey on staff beliefs around CBD at a south London mental health trust. Physicians at different grades, nurses and other healthcare professionals with patient contact were asked questions about their knowledge of and understanding around the benefits and risks of cannabidiol.

The survey assessed agreement levels on statements related to CBD use on the basis of their healthcare roles, as well as their experience of working in a substance misuse unit. The aim was to find out how much awareness clinical staff providing mental health care had about CBD in the UK and to what extent this is related to their profession or having a background in addiction work.

## Method

### Participants

The survey was opened in July 2018 over a 3-week period from July 10th to July 30th, at South West London and St George’s Mental Health NHS Trust. A Mental Health NHS Trust is an organisation that provides mental health services within a specific geographical area. Our trust serves a population of 1.1 million people across several South West London boroughs and employs over 2000 members of staff (https://www.swlstg.nhs.uk/about-the-trust).

We used SurveyPlanet.com to create the questionnaire which was voluntarily and anonymously completed after clinical staff received an invitation to do so by Trust email (which uses Microsoft Outlook™) via our clinical support team (administration). This was sent out to clinical staff at the beginning of July 2018, with a reminder email being sent towards the end of the month. The numbers of physicians sent the survey by emails were estimated at 137 Consultants, 12 Associate Specialists, 112 CT1-3 (Core) Trainees and ST4-6 (Specialty/Higher) Trainees, 52 Foundation Physicians and 6 Staff Grade (Non-training Specialty) physicians from information given by our clinical support team (Staff Grade and Associate Specialist doctors are also known as SAS doctors). This gave a total of 319 physicians to whom the email was sent out to. Hidden within the number of Core Trainees are also General Practitioner (GP) trainees, who are on their way to becoming GPs but do 6-month psychiatric placements during their training.

Consultants are senior physicians who have completed specialist training. The equivalent position in the USA is an ‘Attending Physician’. Associate specialists are also considered senior physicians but they report to Consultants. The roles Core trainees, GP trainees, Specialty trainees, Staff Grades Non-training Specialty and Foundation physicians are junior physician roles. The order of training starts with foundation training, followed by Core training, specialty training and then finishing with a Consultant position. Core trainees and Speciality Trainees would be referred to as ‘Residents’ and ‘Fellows’ respectively in the USA. All the physicians in the survey were in the psychiatry service line.

The numbers of other healthcare professionals sent the survey could not be determined, but the survey was disseminated by leaders of their respective service lines in the Trust. This included nurses and other staff with patient contact (which includes psychologists, healthcare assistants, occupational therapists and social workers). As of 31 March 2019, there were 1718 non-physician healthcare professionals working in the Trust, which, excluding physicians, represents a potential maximum number of healthcare professionals that the survey could have been sent to.

According to the Trust Annual report of 2018–2019 (Beasley 2019), there were 2286 employed staff. Most of them are healthcare professionals. It could be estimated that up to 2000 professionals were eligible to take part in this study.

### Survey materials

Basic demographic data were collected, including the profession of the individual and in which locality they worked. Participants were also asked whether they had previously worked in addictions and if they had experience in prescribing medications. The attitudinal items in the survey are shown in Table 1. For each statement, responders had to choose their level of agreement using a 5-point Likert scale (strongly disagree, disagree, neither agree nor disagree, agree, strongly agree). Staff were also given an opportunity to submit free text responses to the survey.

**Table 1:** CBD survey attitudinal items

(i) I am aware of several potential properties for the clinical use of CBD	
(ii) CBD reduces the likelihood of psychosis	
(iii) CBD has serious side effects	
(iv) I have concerns about patients self-medicating with CBD	
(v) CBD is easily available to buy in high street shops	
(vi) People can become dependent on CBD if used regularly	
(vii) CBD should be prescription only	
(viii) I would prescribe CBD if it were in the British National Formulary (this question was only for those with a licence to prescribe)	

It is important to note for question eight that the question could only be answered by physicians for the most part. Since 1992, in the UK, pharmacists, dentists and other healthcare professionals have been allowed to become independent prescribers with gradual expansion in autonomy in this regard (Cope et al. 2016). However, this is not a widespread practice.

### Statistical analysis

Descriptive and attitudinal data are presented in the form of frequencies and percentages. Relationships of key variables (professional role, experience with addictions, able to prescribe medication) with agreement (levels) on survey items were examined using chi-square tests, with odds ratios (*OR*s) and 95% confidence intervals (*CI*s) presented for (planned post hoc) pairwise comparisons in the case of significant associations. The level of significance was set at *p* < 0.05. All statistical analyses were completed with SPSS statistical software, version 24.0 (SPSS, IBM).

## Results

Responses to the online survey were received from 190 London-based mental health clinical staff members during a 3-week period; 2 failed to provide attitudinal data to statements concerning the use of CBD while another 8 worked roles that had no direct contact with patients and, as such, did not qualify to complete the questionnaire. The final sample therefore consisted of 180 responders. Three quarters of these (135 or 75.0%) completed the survey anonymously. Most respondents completed the survey using a PC (Windows operating system; 166 or 92.2%); 14 (7.8%) responders used a mobile phone (I-phone, Android or Windows). The staff characteristics of responders are shown in Table 2. Of those who responded effectively, 30.5% (55/180) were physicians. Notably, solely based on the number of physicians requested to respond, the response rate was low, with 17.2% (55/319) of physicians responding. The response rate for non-physicians could not be calculated, as their total numbers were not known at the outset.

**Table 2:** Staff profile of CBD survey responders (*n* = 180). Values represent frequencies (percentages)

Role	
Consultant/SAS	29 (16.1)
Nurse	62 (34.4)
CT/GPVTS/FY	17 (9.4)
SpR/ST	9 (5.0)
Other staff with patient contact	63 (35.0)
Experience of working with addictions services/patients	95 (52.8)
Prescribe medications	58 (32.2)
Prescribe medications (and a physician)	55 (30.5)
Proportion of prescribers who are physicians	55/58 (94.8)

*SAS*, Specialty Physicians and Associate Specialist; *CT*, Core Trainee; *GPVTS*, General Practice Vocational Training Schemes; *FY*, Foundation Year Physician; *SpR*, Specialist Registrar; *ST*, Specialty Trainee

### Attitudes concerning the use of cannabidiol (CBD)

The overall agreement levels are summarised in Table 3.

**Table 3:** Attitudes concerning use of cannabidiol (CBD; *n* = 180). Please note that values represent frequencies (percentages)

	Strongly agree	Agree	Neither agree nor disagree	Disagree	Strongly disagree	Overall % agree
I am aware of several potential properties for the clinical use of CBD	21 (11.7)	80 (44.4)	39 (21.7)	28 (15.6)	12 (6.7)	101 (56.1)
CBD reduces the likelihood of psychosis	5 (2.8)	33 (18.3)	62 (34.4)	57 (31.7)	23 (12.8)	38 (21.1)
CBD has dangerous side effects	14 (7.8)	38 (21.1)	81 (45.0)	39 (21.7)	8 (4.4)	52 (28.9)
I have concerns about patients self-medicating with CBD	34 (18.9)	67 (37.2)	60 (33.3)	17 (9.4)	2 (1.1)	101 (56.1)
CBD is easily available to buy in high street shops	19 (10.6)	40 (22.2)	48 (26.7)	53 (29.4)	20 (11.1)	59 (32.8)
People can become dependent on CBD if used regularly	14 (7.8)	55 (30.6)	66 (36.7)	40 (22.2)	5 (2.8)	69 (38.3)
CBD should be prescription only	20 (11.1)	66 (36.7)	53 (29.4)	28 (15.6)	13 (7.2)	86 (47.8)
I would prescribe CBD if it was in the British National Formulary	9 (15.5)	35 (60.3)	10 (17.2)	2 (3.4)	2 (3.4)	44 (75.9)

Note: for questionnaire item ‘I would prescribe CBD if it was in the British National Formulary’, only staff who reported prescribing medication were considered; *n* = 58

### Impact of professional role and experience with addictions on attitudes towards CBD

**Fig. 1:**
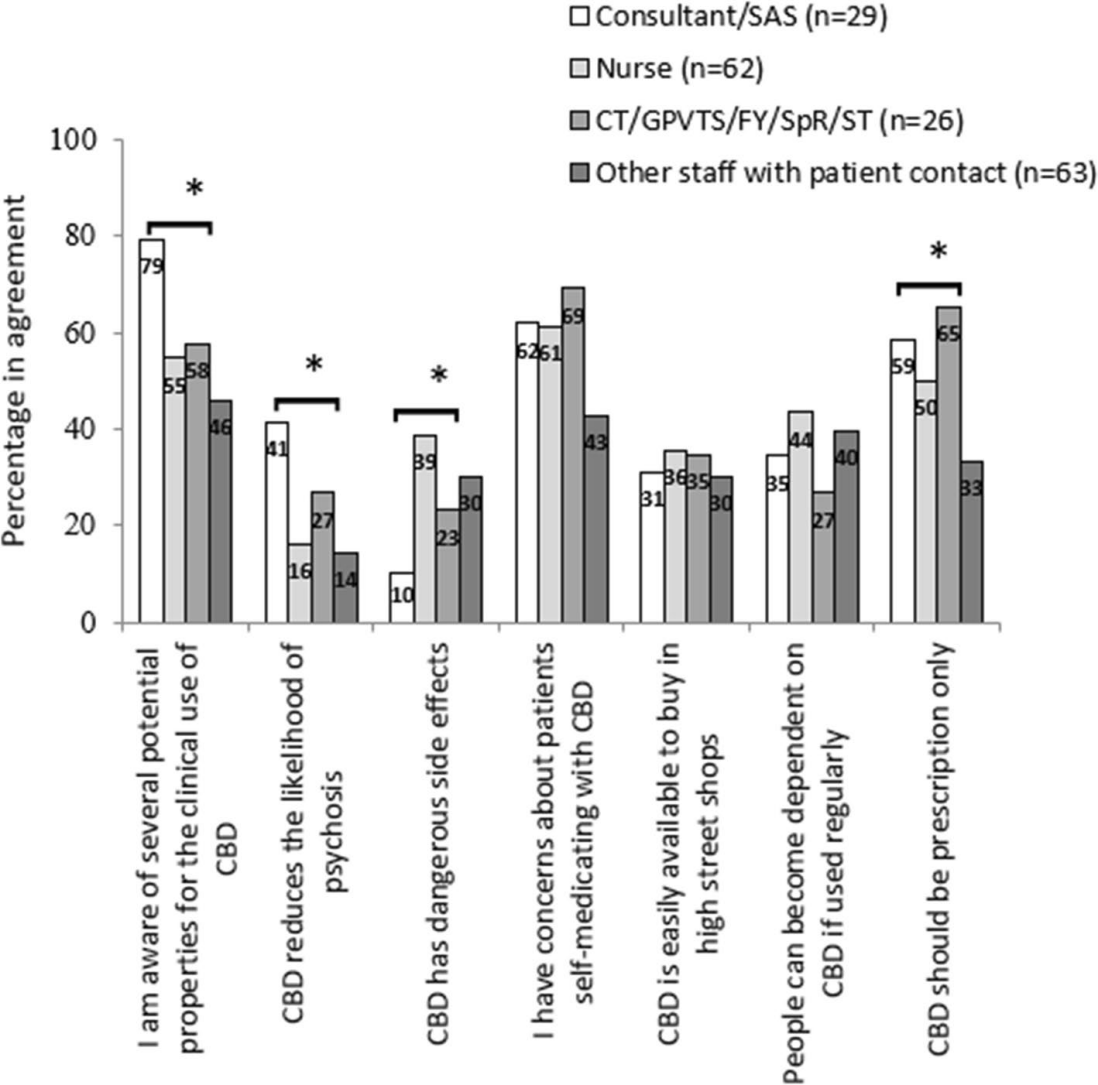
Staff attitudes toward the medical use of cannabidiol, by professional role (percentage responding either ‘agree’ or 'strongly agree’). Data labels represent percentages; differences are on chi-square testing; *p < 0.05

There were differences in attitudinal responses to CBD according to the professional role of the respondents. These were significant on chi-square testing at a significance level of *p* <0.05, on items regarding beliefs of potential benefits of using CBD in a clinical context, CBD reducing likelihood of psychosis, CBD having dangerous side effects and whether CBD should be prescription only (Fig. 1). Generally, for these items, differences were most obvious between physicians (consultants, SAS, junior physicians) and nurses or staff with other patient contact. Post hoc pairwise comparisons confirmed that consultant physicians/SAS demonstrated significantly higher rates of agreement for the belief of potential properties for the clinical use of CBD than nurses (*OR* = 3.16, *CI* = 1.13, 8.83; *p* = 0.025) and other staff with patient contact (*OR* = 4.49, *CI* = 1.61, 12.54; *p* = 0.003). Consultant physicians/SAS also showed greater levels of agreement concerning the statement that CBD reduced the likelihood of psychosis compared to nurses (*OR* = 3.67, *CI* = 1.34, 9.99; *p* = 0.009) and other staff with patient contact (*OR* = 4.23, *CI* = 1.52, 11.77; *p* = 0.004). Significantly fewer consultant physicians/SAS were concerned with dangerous side effects of CBD compared with nurses (*OR* = 0.18, *CI* = 0.05, 0.67; *p* = 0.006) and other staff with patient contact (*OR* = 0.27, *CI* = 0.07, 0.99; *p* = 0.038). Both consultant physicians/SAS (*OR* = 2.83, *CI* = 1.15, 7.01; *p* = 0.022) and junior physicians (*OR* = 3.78, *CI* = 1.44, 9.90; *p* = 0.005) were significantly more likely than other staff with patient contact to agree that CBD should be prescription only. Agreement levels were similar across professional groups for items relating to concerns about patients self-medicating with CBD, whether CBD is easily available at high street shops. Agreement levels were also similar across groups on the risk of patient dependency on CBD.

**Fig. 2:**
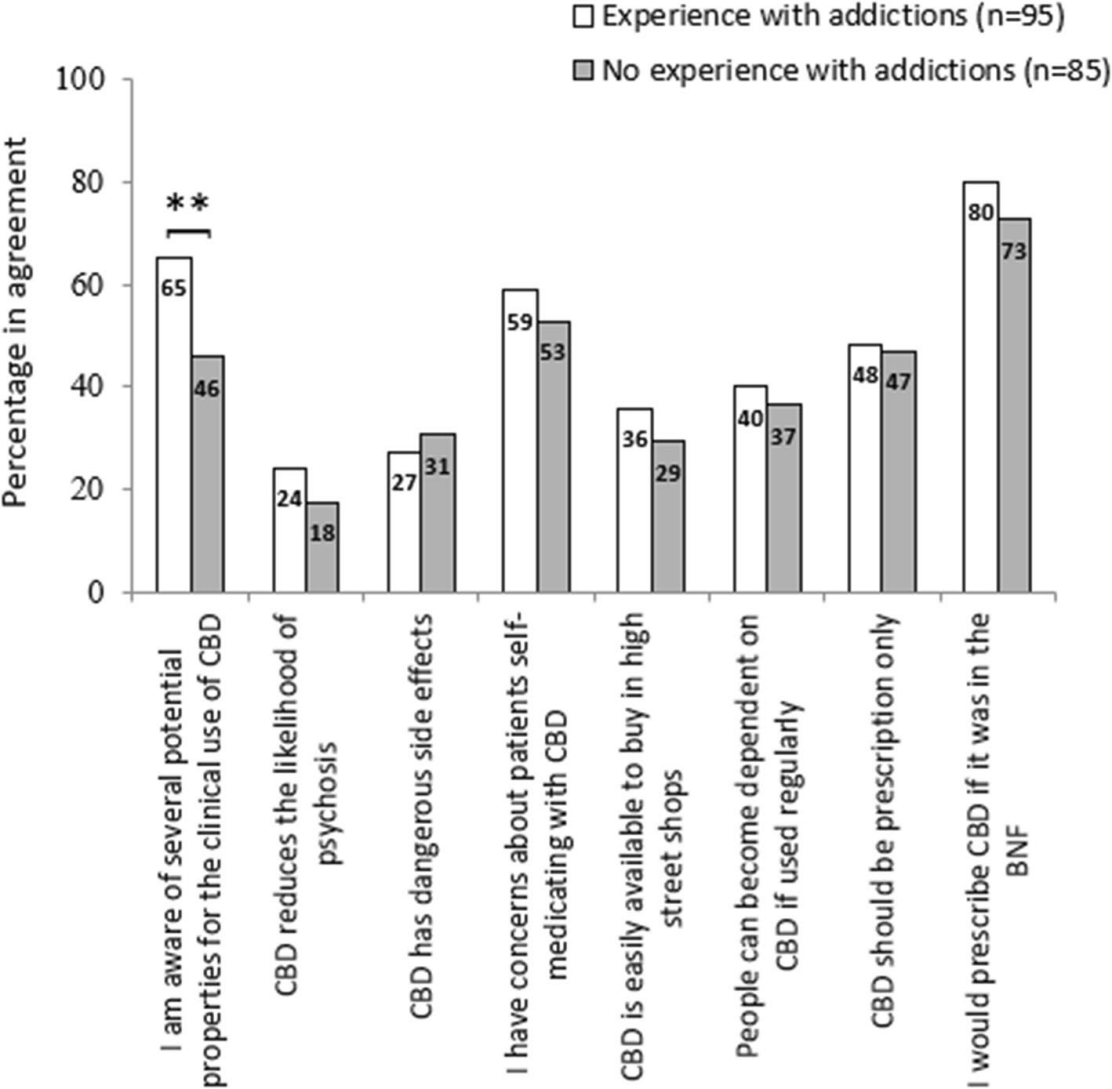
Staff attitudes toward the medical use of cannabidiol, by addiction experience (percentage responding either ‘agree’ or 'strongly agree’). Data labels represent percentages; differences are on chi-square testing; **p < 0.01

In contrast, there were fewer differences in attitudinal responses according to whether individuals had experience of working with addiction services/patients (Fig. 2). Only on the matter of being aware of potential properties for the clinical use of CBD did agreement levels significantly differ. This was significant on chi-square testing at *p* < 0.01. Post hoc pairwise comparison confirmed that individuals with a history in working with addictions were more likely to agree with statements than those individuals without such experience (*OR* = 2.22, *CI* = 1.22, 4.04; *p* = 0.009).

**Fig. 3:**
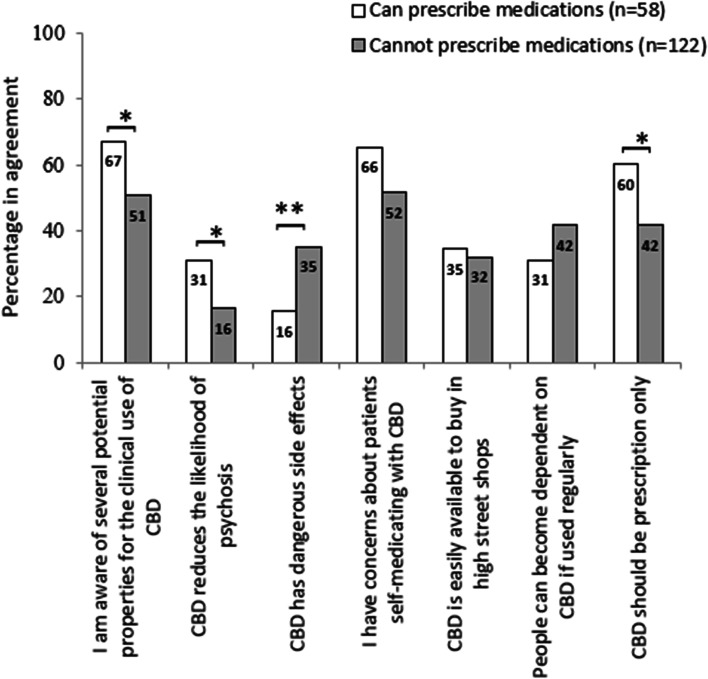
Staff attitudes toward the medical use of cannabidiol, by ability to prescribe (percentage responding either ‘agree’ or ‘strongly agree’). Data labels represent percentages; differences are on chi-square testing; *p < 0.05, **p < 0.01

There were significant differences in attitudes between healthcare professionals with a licence to prescribe and those without in several domains. These domains were potential therapeutic benefits, reduced likelihood of psychosis, dangerous side effects and CBD needing to be prescription only. They were significant at a *p* <0.05 level on chi-square testing, except for ‘dangerous side effects’, which was significant at *p* <0.01. Healthcare professionals with a licence to prescribe were more likely to agree that there are potential properties of CBD for clinical use (Fig. 3) (*OR* = 1.99, *CI* = 1.03, 3.82; *p* = 0.038). They were more likely to agree that CBD may reduce psychosis (*OR* = 2.30, *CI* = 1.10, 4.78; *p* = 0.024) than professionals without a licence to prescribe. They also demonstrated much less agreement with the statement that CBD has dangerous side effects (*OR* = 0.34, *CI* = 0.15, 0.75; *p* = 0.006). Professionals who could prescribe medication were more likely to agree that CBD should be prescription only (*OR* = 2.12, *CI* = 1.12, 4.01; *p* = 0.020).

Here is the selection of free text responses on the survey.

Consultant/SAS: ‘Need more research on CBD’

Nurse: ‘Further research and evidence needed for the clinical use of CBD in mental health setting.’

Other staff with patient contact: ‘I have no information or knowledge of CBD’

Consultant/SAS: ‘Distinction between medicalisation, decriminalisation and legalising cannabis important. CBD needs to go through normal rigorous research trials before we can prescribe. Very serious concerns about legalising cannabis/CBD.’

Other staff with patient contact: ‘I am not very up to date with the effects of Cannabidiol. However, I am extremely concerned that patients of all ages are being admitted to hospital due to psychosis triggered by cannabis. I would really appreciate further training in relation to what is in cannabis and the effects on patients both short term and long term.’

## Discussion

Overall, staff may have demonstrated some awareness of the potential therapeutic benefits of CBD with over 50% agreeing to this statement. However, the significant confound is cannabis. Cannabis is well known for its potential use as medical therapy in conditions such as epilepsy, multiple sclerosis and chronic pain. Participants could have responded in kind on that basis.

Most did not agree that CBD reduces the likelihood of psychosis or that it has dangerous side effects. Multiple considerations can be made from these outcomes. Firstly, although there is literature including randomised controlled trials (RCT) to suggest that CBD can be of benefit in attenuating psychotic symptoms (Iffland and Grotenhermen 2017; Bhattacharyya et al. 2018; Davies and Bhattacharyya 2019), these studies are not necessarily well known. In that vein, the reason why few agreed that CBD has dangerous side effects might not be because they are aware of any literature or media that supports the statement. Rather, it may be that they are not sure at all but expect that if there were they would somehow know about it, or have considered that cannabis in general is not known for having dangerous side effects, albeit for psychotic relapse.

The low agreement with CBD having dangerous side effects and yet over 50% of participants had concerns about self-medicating with CBD could suggest that generally unsure about it and lack up-to-date information. In association, with this, the apprehension about self-medication might not be just about CBD but pre-existing biases about cannabis. Indeed, it is possible this question might have been answered with cannabis in mind rather than CBD.

Overall, less than one-third of participants believed that CBD was available in high street shops. This confirms the general lack of awareness about CBD legislation and, as mentioned earlier, might suggest that the question was answered in the view of cannabis instead.

The concerns about dependence on CBD could again be due to pre-existing beliefs regarding CBD, and the same could be stated for the question of whether it should be prescription only.

Just over 75% of prescribers demonstrated a willingness to prescribe CBD if it was in the British National Formulary. This is in alignment to a degree with the overall consensus that CBD may have potential therapeutic benefits.

It can be considered on reflection that there were key discrepancies across three questions: CBD does not have dangerous side effects, CBD is easily available in high street shops and CBD should be prescription only. Most participants did not believe it had dangerous side effects, were not aware that it is available on the UK high street and were keen for it to be prescription only. This could point towards a general lack of awareness. But this can only be stated cautiously as some of the professionals could have been aware that CBD is available on the high street but still maintain that it *should* be prescription only.

As stated above, there are some signs that participants answered on the basis of cannabis and not CBD, which also highlights a potential lack of awareness.

However, when the results are delineated for job description, it becomes clear that the Consultant and SAS group responses are different to other groups especially nurses and ‘other staff with patient contact’. They were more likely to believe that CBD could have therapeutic property, reduce psychosis and lack dangerous side effects. But this is contrasted by a higher agreement with it being prescription only compared to ‘other staff with patient contact’. This is difficult to explain but it can be surmised that despite an awareness of these factors, physicians may still prefer CBD to be prescription only, or they may have been responding with a hidden cannabis bias. Physicians may also be thinking about possible interactions with other prescribed medications when making their evaluation. One can also suspect the reticence is due to the reasonable opinion that there is insufficient data on the safety and efficacy of CBD. This has been evidenced in a sample of neurologists with regard to medical cannabis and CBD in epilepsy, with the specialist neurologists proving more cautious than other medical professionals and patients (Malthern et al. 2015).

Having a background in addiction service work was a clear signifier that the professional was more likely to demonstrate awareness about the potential therapeutic benefits of CBD. However, as mentioned above, the potential hidden bias of answering the question in the context of cannabis cannot be discounted. Addiction experience made no difference to whether the professional was likely to believe that CBD should be prescription only, and absolute agreement levels were high regardless.

The significant agreement differences to the questions on basis of ‘ability to prescribe’ map closely to those found on basis of the job description. This is immediately intuitive when it is considered that 94.8% of those able to prescribe in this study are physicians. The key difference is that with non-prescribers that belief of CBD having dangerous side effects takes on a greater significance.

It is unclear if physicians respond differently due to more knowledge about CBD, although due to the expected training regimen and the expectation to deal with medications, it is expected that physicians should know more about pharmacological substances compared to other healthcare professionals.

Unfortunately, there were few free text responses but those given may reveal some of the considerations that have already been made. A Consultant/SAS response stated serious concern about legalising cannabis and CBD, making no distinction between the two. A member of staff who was neither a nurse or physician stated overall concern secondary to psychosis triggered by cannabis, stating a need to know more about what is in cannabis. Both responses highlight that many participants may have responded to questions on the basis of cannabis as opposed to CBD. The responses also indicated the subjective lack of knowledge participants had. And free text response by a nurse highlighted the need for more CBD research in mental health settings.

There is evidence that suggests CBD may have a therapeutic effect on psychosis (Iffland and Grotenhermen 2017; Bhattacharyya et al. 2018). There is no evidence in the literature at present to suggest that CBD worsens psychosis. This is in stark contrast to THC, which is associated with increased psychosis (Di Forti et al. 2009). While there was some awareness of it being potentially beneficial therapeutically, even among physicians, the awareness that it could reduce the likelihood of psychosis was low. But this could have been due to how the statement was phrased. Stating that ‘CBD *may* reduce the likelihood of psychosis’ would have been more precise, although one could also argue that using the qualifier of ‘may’ would lead the respondent towards agreement automatically.

On the issue of the questions asked, it would have helped to have asked a greater breadth of statements to tease out other factors that have not been captured. Two examples would be statements to the effect: (1) CBD and cannabis have the same effects and (2) CBD can interact with other medications. Statements like these could have aided us in considering why staff in general scored low on agreement with CBD potentially reducing psychosis and sustained high agreement levels for worries about patients self-medicating with CBD and CBD being prescription only.

Up until recently, CBD had not been associated with severe side effects and has been thought of as a potential adjunctive treatment for that very reason (Iffland and Grotenhermen 2017). Its common side effects were noted as tiredness and diarrhoea (Iffland and Grotenhermen 2017). However, since 2018, the incidence of adverse drug reactions has increased significantly as per figures from VigiBase® (the World Health Organization’s global database for adverse drug reactions: www.vigiaccess.org). Healthcare professionals who were consultants or junior physicians were less likely to think it did have severe side effects. However, the reality is that it is still not clear how much CBD is associated with severe side effects.

CBD could technically have interactions with antidepressants (SSRI, tricyclics) and benzodiazepines. CBD inhibits and is metabolised by the CYP3A4 enzyme (Iffland and Grotenhermen 2017; Watanabe et al. 2007; Yamaori et al. 2011) and inhibits CYP2D6 (Aso et al. 2019), so its levels would be affected by inhibitors or inducers of these enzymes and it can also affect the metabolism of other drugs. CYP2D6 inhibition could increase the concentration of SSRIs and antipsychotics while CYP3A4 inhibition can increase the serum concentration of benzodiazepines. So, it is reasonable to consider that concerns about interactions with other drugs are relevant with regard to CBD.

Current research has not associated CBD with addictive potential and there is some evidence for it being considered as a form of treatment for addiction disorders (Prud’homme et al. 2015).

To our knowledge, our study is the only survey of its kind testing attitudinal differences specifically in regard to CBD and with particular regard to psychiatric symptoms (e.g. psychosis). There are similar studies for medicinal cannabis.

A study of a Minnesota (USA)-based health system (where there has been a medical cannabis system since 2014) found that a majority of healthcare professionals surveyed (76% physicians) were in favour of cannabis as medical therapy (58.1%). This is comparative with the agreement levels for potential therapeutic benefits of CBD being at 56.1% in our study. This study had a measured response rate of 31% but only had 62 completed surveys compared to our 180 (Philpot et al. 2019).

An Australian study of psychiatrists and psychiatry trainees only, across several different territories, used a much broader 64 items in its survey compared to our 8 items. Fifty-five physicians completed the survey, yielding a response rate of 1.1%, with over 66.7% of respondents believing that CBD has therapeutic use for childhood epilepsy, chronic pain, nausea and vomiting (Jacobs et al. 2019). This seeming overall agreement of potential benefit from CBD was a characteristic shared by our studies. Just like our study, these studies also noted a likely lack of knowledge on the part of the healthcare professionals that would benefit from education.

It is fair to state that there are notable differences in attitudes and beliefs of different healthcare professionals with respect to CBD. From the responses given, it can be inferred that there is also a lack of knowledge about it and staff could benefit from some form of education.

Despite this, it is also important to consider that there is still a lack of established information on CBD potential uses, efficacy and safety, although much of the research to date has been promising. This is a significant factor in this survey and the mixed nature of the results may also reflect this.

### Limitations

Apart from nurses, the non-physicians in the survey were treated as one group, which led to a loss of detail in the responses. Pharmacists handle medication and are likely to have more awareness about CBD than psychologists for instance. For future study, the service lines of non-physicians would have to be delineated.

Limiting the number of questions in a survey may encourage participation, as it appears less time consuming. However, the set of CBD-related statements employed in the survey were limited in scope and a larger set of assertions could have been used to allow for more insight into participants’ opinions.

Sampling was not randomised in any way and the study sample was a convenience one. This exposes our study to sampling bias as it may overrepresent staff who have strong feelings regarding cannabis and CBD and therefore be more likely to complete the survey.

Also, studying a single mental health trust in one city in the UK limits generalisability to the whole healthcare population. A wider study across several mental health trusts in the UK would improve the prospect of external validity.

## Conclusion

From this evaluation of survey responses, it appears that mental health clinical staff in the UK require more awareness about the availability and potential uses of CBD especially given the recent changes in the law. This would enable them to better counsel patients about CBD, especially considering the growing availability of the substance without prescription will likely lead to more patients presenting to mental health services as users of CBD. Given these factors, it seems beneficial for mental health trusts in the UK to provide more training so that staff feel confident in making decisions regarding CBD.

## Data Availability

Available upon request

## Abbreviations

CBD: Cannabidiol
THC: Tetrahydrocannabinol
CYP3A4: Cytochrome P450 3A4 enzyme
CYP2D6: Cytochrome P450 2D6 enzyme
*OR*: Odds ratio
*CI*: Confidence interval
SSRI: Selective serotonin reuptake inhibitor
SAS: Specialty Physicians and Associate Specialist
CT: Core Trainee
GPVTS: General Practice Vocational Training Scheme (GP trainee)
FY: Foundation Year Physician
SpR: Specialist Registrar (equivalent to ST)
ST: Specialty Trainee (equivalent to SpR)
CPD: Continuous professional development
BNF: British National Formulary
